# An exploration of the experiences and self-generated strategies used when navigating everyday life with Long Covid

**DOI:** 10.1186/s12889-024-18267-6

**Published:** 2024-03-13

**Authors:** Fiona J. Leggat, Celayne Heaton-Shrestha, Jessica Fish, Aloysius Niroshan Siriwardena, Anne Domeney, Carol Rowe, Ian Patel, Judith Parsons, John Blair, Fiona Jones

**Affiliations:** 1 Population Health Research Institute, St George’s, University of London, London, England UK; 2 Centre for Applied Health and Social Care Research, Faculty of Health, Social Care and Education, Kingston University, London, England UK; 3 Department of Clinical Neuropsychology & Clinical Health Psychology, St George’s University Hospitals NHS Foundation Trust, London, England UK; 4 Mental Health & Wellbeing, School of Health and Wellbeing, University of Glasgow, Glasgow, Scotland UK; 5 Community and Health Research Unit, University of Lincoln, Lincoln, UK; 6 LISTEN Lived Experience Advisory Group, London, UK; 7 Bridges Self-Management, London, England UK

**Keywords:** Long Covid, Self-management, Lived experience, Narrative, Qualitative research, Covid-19

## Abstract

**Background:**

Around one in ten people who contract Covid-19 report ongoing symptoms or ‘Long Covid’. Without any known interventions to cure the condition, forms of self-management are routinely prescribed by healthcare professionals and described by people with the condition. However, there is limited research exploring what strategies are used to navigate everyday life with Long Covid, and experiences that initiate development of these strategies. Our study aimed to explore the range and influence of self-generated strategies used by people with Long Covid to navigate everyday life within the context of their own condition.

**Methods:**

Forming part of the Long Covid Personalised Self-managemenT support co-design and EvaluatioN (LISTEN) project, we conducted a qualitative study using narrative interviews with adults who were not hospitalised with Covid-19. Participants aged over 18 years, who self-identified with Long Covid, were recruited from England and Wales. Data were analysed with patient contributors using a reflexive thematic analysis.

**Results:**

Eighteen participants (mean age = 44 years, SD = 13 years) took part in interviews held between December 2021 and February 2022. Themes were constructed which depicted 1) the *landscape behind the Long Covid experience* and 2) *the everyday experience* of participants’ Long Covid. The everyday experience comprised a combination of physical, emotional, and social factors, forming three sub-themes: *centrality of physical symptoms, navigating ‘experts’ and the ‘true colour’ of personal communities,* and *a rollercoaster of psychological ambiguity*). The third theme, *personal strategies to manage everyday life* was constructed from participants’ unique presentations and self-generated solutions to manage everyday life. This comprised five sub-themes: *seeking reassurance and knowledge, developing greater self-awareness through monitoring, trial and error of ‘safe’ ideas, building in pleasure and comfort,* and *prioritising ‘me’*.

**Conclusions:**

Among this sample of adults with Long Covid, their experiences highlighted the unpredictable nature of the condition but also the use of creative and wide ranging self-generated strategies. The results offer people with Long Covid, and healthcare professionals supporting them, an overview of the collective evidence relating to individuals' self-management which can enable ways to live ‘better’ and regain some sense of identity whilst facing the impact of a debilitating, episodic condition.

**Trial registration:**

LISTEN ISRCTN36407216.

**Supplementary Information:**

The online version contains supplementary material available at 10.1186/s12889-024-18267-6.

## Introduction

In 2021, the World Health Organisation (WHO) officially recognised the complex, and persisting symptoms experienced by individuals after contraction of the Covid-19 virus [1]. Termed Post Covid-19 syndrome by WHO, various names have been given to the condition including ongoing symptomatic Covid-19, post-acute Covid-19, and Long Covid [1, 2]. With ‘Long Covid’ collectively used by the community of people experiencing the condition [3], the term has now been widely adopted.

In the UK, current estimates from early 2023 suggest that approximately 1.9 million people are experiencing Long Covid [4]. The potential burden of Long Covid is substantial with 79% people reporting adverse impact on day-to-day activities [4]. Many people experience prolonged symptoms and an absence from work for over six months [5]. However, figures are likely an underestimate given the absence of testing early in the pandemic and with an absence of accurate or consistent data on the condition collected from NHS England, self-report data is still relied upon [6]. Since 2023 the public spotlight has reduced considerably, and UK government rarely report figures for Long Covid in national media. Yet, consistent with past data on prevalence [7], estimates suggest Long Covid is not going away, with over 40% of those reporting Long Covid infected at least two years previously [4]. In addition, given that Long Covid is most prevalent in the working age population (e.g., 35–69 years) [4], the impact on the UK economy is substantial.

Symptoms of Long Covid are wide-ranging and fluctuating [8]. Over 200 symptoms across 10 organ systems have been identified [5], with fatigue and breathlessness most common [4]. Those living with Long Covid have described distinctive pervasive neurocognitive and physiological symptoms, and the interaction and impact of symptoms [9–11]. The severity and unpredictable nature of symptoms can contribute to multiple psychological and social effects [12]. People with Long Covid have described ‘spoiled’, ‘altered’ and ‘diminished’ identities, unable to fulfil prior responsibilities (e.g., employment, family roles, personal care). They have described experiencing guilt, stigma, and shame that they developed Long Covid and cannot do what they once did [12–14]. For some, the impact is heightened by the ‘invisibility’ of symptoms [11], and compounded by a lack of validation and understanding from others [14, 15]. This has led to many individuals feeling unsupported and helpless, which can adversely affect social relationships, ability to work and accelerate feelings of isolation [12, 13, 15–17]. Some have referred to Long Covid as an “end to normality” [15].

For people with Long Covid living in England, NHS commissioning guidance for post-Covid services includes the provision of self-management support, multidisciplinary rehabilitation, and specialist referral [18]. However, provision and access to services is variable, ranging from referral to one of 90 specialist Long Covid clinics, to use of digital platforms such as Your COVID Recovery or Living with Covid Recovery [19, 20]. Research has further highlighted that people with Long Covid may lack trust in healthcare professionals and avoid services because of some professionals’ disbelief and lack of knowledge about the condition [17]. To date, there is no single intervention for those living with Long Covid which has shown evidence of effectiveness, and the underpinning evidence and rationale of current NHS services is unclear.

With the contrasting provision and absence of support, people have engaged in self-discovery and turned to the collective knowledge and creativity of the Long Covid community for answers (e.g., internet, Long Covid support groups) [15]. Strategies reported have included energy moderation (e.g., pacing, prioritising, taking breaks), diet control (e.g., supplements, eating healthily), pharmacological treatments (e.g., over-the-counter medication), and distraction (e.g., meditating, being outside) [11, 14, 15, 21]. For some, one or a combination of these have offered relief for specific symptoms, yet for others, there has been little to no benefit [11, 14]. One study explored the impact of a self-management programme on the wellbeing of people with Long Covid [22]. Although findings suggested an increase in wellbeing during the intervention, it is not known what information and strategies from the programme were used by participants, nor which may have had an impact [22].

Overall, national provision for people with Long Covid in England and Wales includes self-management support, yet little is known about the efficacy of such programmes and the quality of advice provided. With the collective trust and growing confidence people with Long Covid have in their own community, this study aimed to explore the range of self-generated strategies used by people with Long Covid to navigate everyday life, the influence of these strategies within the context of their own condition and how they are experienced within the evolving landscape of Long Covid in England and Wales.

## Methods

### Context and research design

**Fig. 1:**
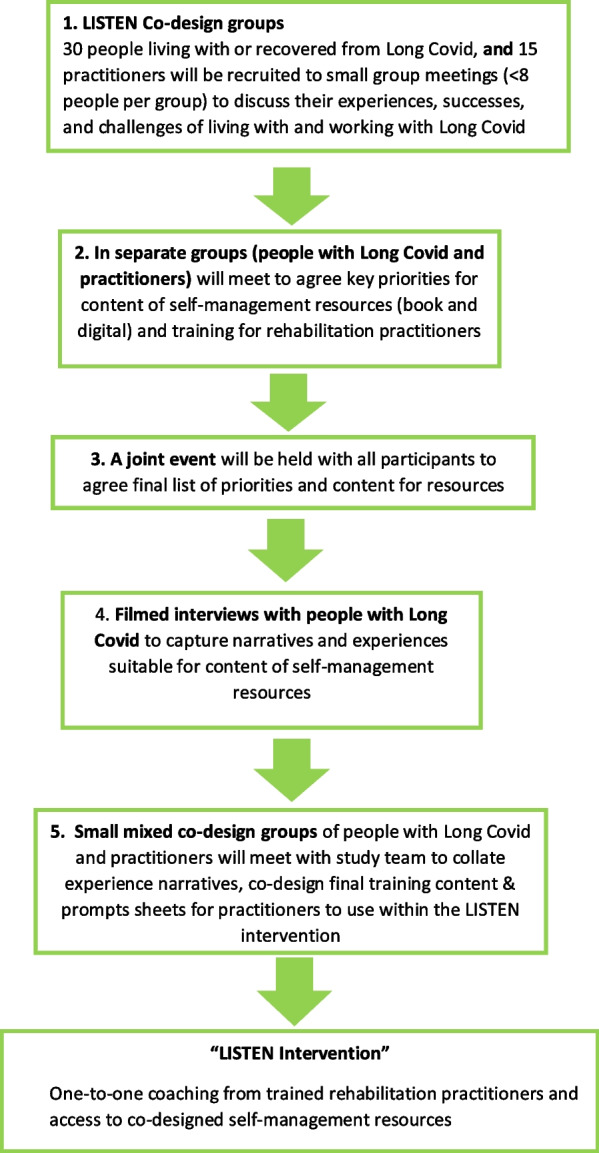
Co-design process flowchart. Reprinted with permission from [26]

In July 2021, the Long Covid Personalised Self-managemenT support co-design and EvaluatioN (LISTEN) project was launched to co-design and evaluate an intervention for people with Long Covid [23]. The first phase of the project adopted a multi-phase, co-design approach using an accelerated form of Experience-Based-Co-Design (AEBCD), based on participatory methods (see Fig. 1) [24, 25]. By harnessing the priorities, ideas, and solutions of people living with Long Covid, LISTEN sought to address the gap that can exist between interventions and the people they seek to support. The collaborative process involved gathering lived experiences of people with Long Covid through focus groups and interviews, summarising key themes, and subsequently using these to trigger discussions about priorities for the intervention. During the process, co-design activities, including narrative interviews, were used to explore peoples’ lived experiences of Long Covid, including everyday challenges, strategies developed and tips for others [26]. This paper presents the analysis of findings from the narrative interviews conducted as part of the co-design process.

### Ethics and governance

Institutional ethical approval was granted on 10th September 2021. Consent to take part in the co-design process was sought via email prior to co-design participation [26]. The co-design phase of the LISTEN project received support from a patient and public advisory group and was overseen by an independent steering committee. Five individuals living with Long Covid were co-authors and worked on the development of this paper.

### Participants

Narrative interview participants were purposively selected from the wider LISTEN co-design group who had consented to take part [26]. The recruitment strategy (e.g., social media) and inclusion criteria (e.g., not hospitalised with Covid-19) are described elsewhere [26]. Several sampling criteria were employed to ensure a variety of participant experiences were collected. These included sociodemographic factors (e.g., age/gender/ethnicity) and the range of Long Covid symptoms, severity, and the phase of the Long Covid ‘journey’^1^ [e.g., recently developed/diagnosed with Long Covid, been living with the condition for some time, recovered]).

### Narrative interviews

Consistent with a narrative approach, interviewees were invited to tell their story, uninterrupted, using their own words. Participants were first asked to describe themselves and their lives, before exploring their Covid-19 and Long Covid experiences. Interviewers used conversational prompts to keep the narrative flowing (e.g., “what happened next?”). Additional open questions were used to gain a deeper understanding of their challenges, personal strategies and tips they had for others, and experiences with healthcare professionals, family, and friends [26] (see Supplementary Material 1: Appendix 1).

Narrative interviews took place between December 2021 and February 2022. All data were collected using Zoom (the platform used for the co-design meetings), although telephone conversations were also offered. In recognition of participants’ symptoms, steps were taken to enable inclusion (e.g., interview topic guides were sent in advance, participants given opportunities to take regular breaks or turn cameras off). Interviews were undertaken by FL, CHS and FJ who had prior experience with qualitative interviewing, and familiarity with Long Covid from the co-design process. Interviews lasted between 32 and 122 min (*M* = 54, *SD* = 19), were audio recorded, and transcribed verbatim.

### Data analysis and methodological rigor

A reflexive thematic analysis (TA) was used to construct themes from the interview data [27, 28]. Underpinned by an epistemological constructionist paradigm and drawing from the tripartite typology for classifying forms of TA [29], a reflexive (Big Q) approach was used. Reflexive TA was sought to enable the inductive exploration of data and facilitate the crafting of themes through researcher interpretation and pre-existing knowledge from the wider LISTEN co-design process. The TA process involved multiple fluid phases intertwined with techniques to enhance the study’s methodological rigor [27, 28, 30] (see Table 1).

**Table 1:** Overview of the Thematic Analysis (TA) process informed by Braun & Clarke [27–29]

Reflexive TA phase	How was the phase actioned? Why was it actioned in this way?
**1. Transcripts were read and re-read by authors**	Transcripts were uploaded to Nvivo Windows (Release 1) and read independently by each author for familiarisation Authors were encouraged to be reflexive through critically self-evaluating their reasoning, positionality, and influence on the interpretation of research (e.g., “what about me is influencing my interpretation?”)
**2. Authors coded eight transcripts**	Codes consistent with the study aims were constructed within NVivo in a pluralistic fashion. Two forms of coding were used relating to the narrative structure of the Long Covid stories and the “whats” of the story content. Narrative structure codes were considered those important to a story’s plot line (e.g., timing of Covid-19 infection/realisation of long Covid), and content codes embodied the essence of an experience/strategy [31] Codes were discussed between authors until consensus reached. New codes were crafted where appropriate. Codes were used as a loose guide for further inductive and deductive coding
**3. Authors independently clustered codes to develop interpretive themes**	Themes were independently developed from initial codes through a fluid process which included re-reading transcripts. Themes were discussed and shared amongst other authors who acted as ‘critical friends’ using Google Jamboard, an online whiteboard platform. Through a collective back and forth, an agreed upon set of initial themes were crafted
**4. Themes developed and refined**	Authors independently returned to the transcripts to establish if the collective themes and interpretations of the data were represented within the raw data. Collectively, authors discussed their interpretations of the themes and the complexities to refine further
**5. Interpretations obtained from people with Long Covid**	Reflections from five patient co-authors with Long Covid were sought to explore the trustworthiness in authors’ interpretations, and to provide further richness and depth to understanding. Following this, themes were refined, and shared with the group for further reflection
**6. Results section write-up**	Themes continued to be shaped during the write up of the results. Patient co-authors with Long Covid were involved in the write up of the results section

To enhance the study’s rigor, the quality of the study was considered using the following indicators [30, 32]: merit of the topic (e.g., is this topic significant or of value?”), credibility (e.g., “do thick description and rich quotations used to illustrate findings?”), transparency (e.g., “have the authors clearly outlined the methodological processes followed?”), coherence (e.g., do methods and procedures used align with the intended aim of the research?”) and naturalistic generalisability (e.g., “do findings resonate with outsiders to the research?”). Aligning with these indicators, FL, CHS and FJ shared debated initial interpretations with each other, acting as ‘critical friends’, and gained member reflections from JF, AS and patient co-authors who encouraged further interpretation debate amongst the group (see Table 1). Following main analysis, external reflections were sought from the wider LISTEN trial team not involved in the interviews or co-design phase, as well as healthcare professionals with experience of working with people with Long Covid.

## Findings

Eighteen participants from the co-design activities took part in narrative interviews. Demographic details of participants are presented in Table 2.

**Table 2:** Demographics of interview participants

	**Frequency count**
**Age**	
*18–25*	1
*26–35*	4
*36–45*	5
*46–55*	5
*56–65*	2
*66* +	1
**Gender**	
*Male*	6
*Female*	12
**Ethnicity**	
*White British*	9
*Indian*	1
*Mixed race – Indian & White British*	1
*Mixed race – Black African & White British*	2
*Not reported*	5
**Time spent living with Long Covid**	
*Less than 6 months*	1
*7–12 months*	3
*13–18 months*	1
*19 months* +	8
*Not reported*	5

**Fig. 2:**
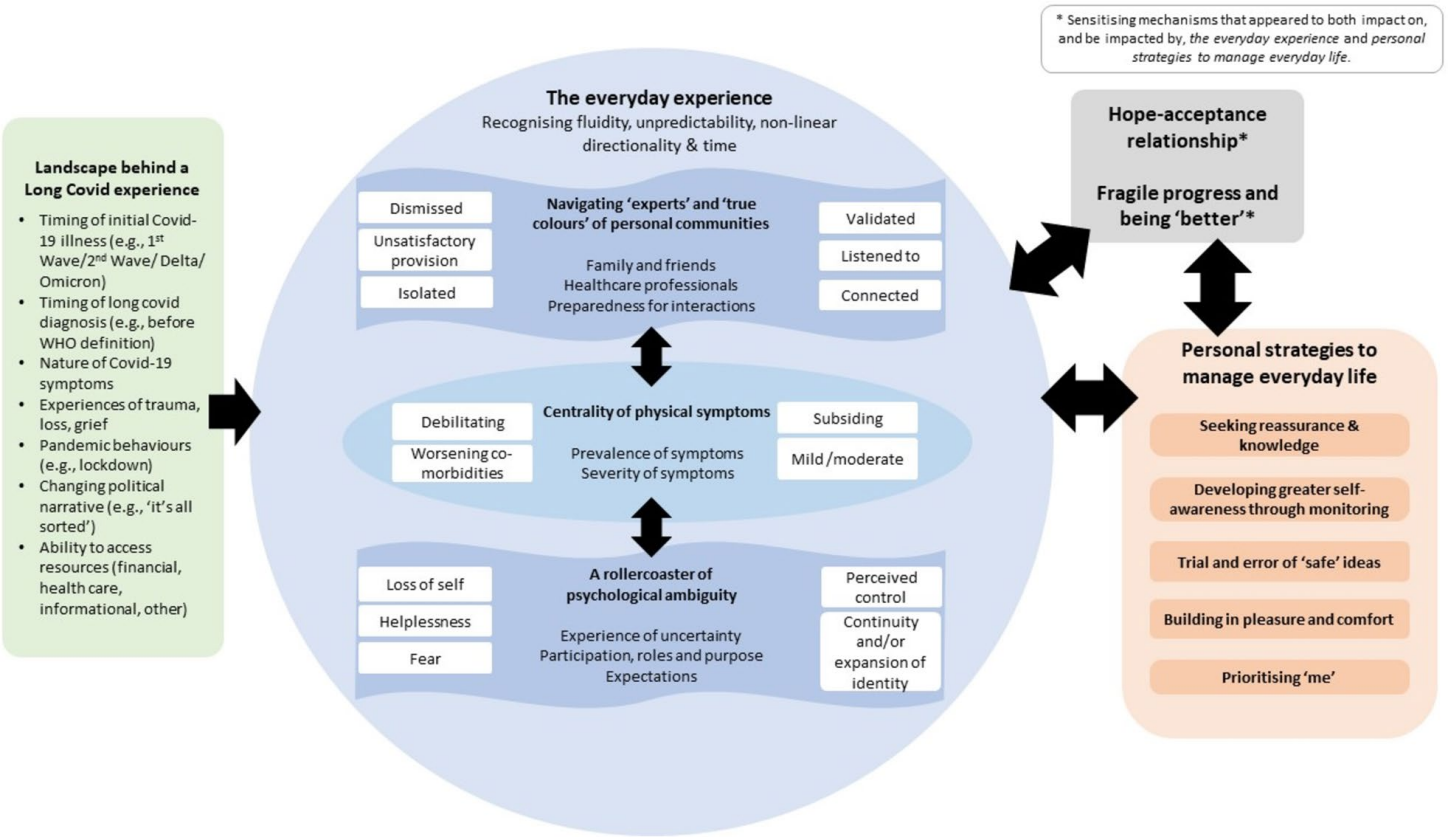
Themes and interactions between themes regarding experiences of and strategies for living with Long Covid

Three themes were constructed from the analysis: 1) *the landscape behind a Long Covid experience*, 2) *the everyday experience,* and 3) *personal strategies to manage everyday life*. Highlighting the complexities surrounding Long Covid, these themes portray depth and intricacy as well as interconnectedness, and are depicted in Fig. 2. The visual representation and framing of the themes and sub-themes were created through discussion with all authors. This has been included to enable readers to make sense of their own, or others’, Long Covid experiences, and reasons behind some of the personal strategies described.

### The landscape behind a Long Covid experience

Stories we tell are not only personal, but socio-contextual constructions. This theme illustrated how the UK pandemic context, including the past and present evolving political and public narrative, influenced participants’ experiences.

Participants’ stories began with ‘unwelcome’, ‘traumatic’ Covid-19 symptoms, and the impact of pandemic restrictions on their lives. Symptoms were ‘mild’, for some, but utterly debilitating for others. Although participants were never hospitalised for Covid-19, some believed they would have been admitted to emergency care outside of the pandemic due to their frightening symptoms. P13 stated “I would go to sleep, and I didn’t know if I was going to wake up”. With peaking health service pressures, participants feared for themselves and their personal communities (e.g., family/friends) as society faced the collective unknown. With imposed government guidelines restricting socialising and group activities, feelings of isolation and a loss of control were prevalent.

Early in the pandemic, blame and stigma were perceived to be attached to contracting Covid-19. Despite obeying government rules and doing everything to stay ‘safe’, some participants were made to feel that catching Covid-19, and Long Covid, was their fault. Although the blame culture changed with vaccine availability, and ‘herd protection’, at the time, the fault placed upon individuals and their failed responsibility prompted feelings of intense frustration about their illness:

Everybody is coming to Long Covid with the context of the pandemic and experiences that are hugely different. A proportion will be coming with the view that there’s been action or inaction through Government and policies, that’s meant that this has happened to them, and it shouldn’t have…I didn’t go to a nightclub or meet up with anyone. I did everything right and I still got it. (P12)

Usual rules appeared not to apply. Conforming to societal norms (e.g., keeping fit and healthy) and obeying government regulations offered little protection. Despite media and government fostering a sense that healthy, young individuals would ‘recover’, participants described the confusion when symptoms persisted or re-emerged weeks later. The realisation or diagnosis of Long Covid constituted a strange period of individual ‘limbo’. With traditional protective factors (e.g., demographic/medical) not applying, P8 described it as a “waiting game”, while P2 struggled to understand what was happening:

I spent 3 weeks in bed thinking, ‘why is everybody else getting better and why am I not?’…I was fit and healthy. I had no idea what Long Covid was, people would just keep asking, ‘when are you going to get better? Why are you still feeling like this?’ and I would just say, ‘I don’t know’.

The growing community of individuals experiencing Long Covid brought recognition of the condition and raised public awareness. Appearing in mainstream media and social media channels, participants described ongoing “do I or don’t I have Long Covid” internal dialogues. Awareness appeared to bring reassurance (e.g., that the condition was real) but fear (e.g., that few were recovering). Despite greater attention throughout 2021 and 2022, obtaining Long Covid diagnoses were bewildering for most participants, unable to determine if symptoms were new or exacerbated by existing co-morbidities:

I didn’t think it was Long Covid, I thought it was over-training because the fever and joint pain had resided by then. There was no reason to think that [Covid] was causing the problem… It’s not until the relapse that I’ve been like, whatever that issue was, it’s still very much here. My symptoms were worse than when I originally had them. (P5)

Participants perceived the ebb and flow of government and media attention to be influential on the ongoing public narrative surrounding Long Covid. Participants spoke of fear and confusion in 2020, but moments of relief during 2021, which brought attention and power to the Long Covid community. However, by early 2022, much of the UK was perceived to have moved on, prompting a sense of abandonment, and feeling forgotten. With Covid considered ‘gone’, participants felt healthcare professionals and the public misunderstood Long Covid:

When people say, ‘oh I feel tired too’, I think that’s because they’re not going out every day and things are different. First you have to say, yes, it’s different but my different is even more different from yours because cognitive dysfunction and fatigue are not quite the same as ‘oh I’ve watched so much Netflix my brain’s fallen out’. It’s not the same (P12).

Overall, this theme depicts how participants’ Long Covid ‘journeys’ started with heightened confusion, exhaustion, loss, and isolation, exacerbated by the evolving social context. The impact of governmental policies and media was regarded as ‘gone, but not forgotten’, and still an ongoing influence on day-to-day experiences.

### The everyday experience

This theme depicted the reciprocal, interconnected relationships between physical symptoms, and the social and psychological day-to-day experiences. Continuums were used to illustrate the constant fluctuations and how these impacted on the use of strategies to navigate life with the condition. Three subthemes comprised the everyday experience.

#### Centrality of physical symptoms

Physical symptoms were strongly emphasised as the underlying causes to daily physical, psychological, and social challenges. These included, but were not limited to, debilitating fatigue, breathlessness, chest pain, cognitive difficulty (e.g., memory, processing), dizziness, gastric symptoms (e.g., allergies), headaches, pain, rashes, sleep disturbances, and speech impairments. The unpredictable fluctuating nature of symptoms were integral to these challenges:

The scary thing about Long Covid is it keeps developing. For me it was like ping pong all over my body, one minute it’s in my chest, one minute it’s in my muscles, one minute it’s in my gut… it just keeps moving around the body and sometimes it’s better and sometimes it’s worse. (P14)

When explaining symptoms, participants used comparative language, metaphors, and everyday analogies, seemingly to highlight disparities in their ability and compare from past to present. As the quote from P4 illustrates, the use of familiar, recognisable examples was utilised to help illustrate the magnitude of symptoms and impairment to friends and family:

This theory of a battery life of a mobile phone resonated with me. Every day you go to bed, you plug your battery in, and it recharges and then in the morning you use the energy you have in that battery. For me, that theory failed to explore that we don’t wake up with 100% charge. I relate it to an old Nokia phone you have in the back of your drawer somewhere. If you tried plugging that in now, you’d be lucky to get 20% and it would be on charge all night.

Physical symptoms were considered the eye of the storm that created rippling effects on social and psychological wellbeing. Trying to “push through” inevitably exacerbated these symptoms.

#### Navigating ‘experts’ and the ‘true colours’ of personal communities

Encounters with others (e.g., friends, family, colleagues, healthcare professionals) presented potentially rewarding, but also challenging situations. From participants’ stories, it appeared that Long Covid uncovered the ‘true colours’ of those around them. Assuming those closest to them would understand, they spoke of surprise when some stepped up to fulfil supportive roles, and others remained absent. Support from close social ties could feel comforting and validating, but the invisibility of the condition was considered challenging. An inability to visually evidence symptoms, coupled with minimal energy and word-finding difficulties to explain, all contributed to what felt to be insurmountable misunderstanding from others which was difficult to cope with:

I look fine, so everyone’s like, “Oh, you’re totally better now, why aren’t you doing things?”, which mentally makes you feel awful because it’s taking every ounce of energy in you to just be up… I’ve lost my friends because I don’t feel like anyone understands, it’s like, “Oh, pull yourself together”. (P11)

Misunderstanding extended to interactions with employers, and without a Long Covid ‘end date’, participants found it challenging to explain when they would be able to work, and why a graded return may not be appropriate.

Interactions with healthcare professional ‘experts’ (e.g., GPs, therapists, specialist consultants) carried substantial influence on participants wellbeing. Portraying a desperation for medical support, participants described “going round the houses”, doing everything possible to source helpful medical advice. Whilst obtaining tests and appointments were energy-demanding investments, formal diagnoses opened doors to services (e.g., NHS Long Covid clinics). However, with overwhelming expectation placed on Long Covid services, when services did not meet such expectation, feelings of isolation, helplessness, and frustration were intensified. Expectations could be crushed when symptoms were attributed to other factors besides Long Covid, such as gender (e.g., “the menopause”) or age (e.g., “getting old”), and when healthcare professionals did not believe them or trust participants to know their own bodies, despite a deep-rooted sensation that something was wrong:

I contacted the GP to say I was still out of breath, and she basically thought I was making it up. They said, ‘you didn’t have Covid, it’s all in your head, so go away’. I wrote to the GP again and in the end, they said, ‘okay, we will refer you to a respiratory consultant’. I saw a respiratory doctor and he said, “there’s nothing wrong. I don’t even think it’s anxiety, I think you’re just obsessed with Covid-19 and you need to get over it and move on” ... I came home and cried because I just thought, I know there’s something wrong with me, I can’t breathe. (P17)

Preparedness for seeing healthcare professionals (e.g., gathering notes from past appointments, developing phrases to describe symptoms) resembled a double-edged sword. Without being prepared (e.g., reacting in the moment), participants felt ill-equipped to adequately convey themselves, and felt opportunities for support were wasted. Alternatively, appearing ‘too prepared’ could fuel disbelief and feelings of guilt for wasting NHS resources. For instance, when dressed neatly and with prepared notes, professionals considered participants ‘too well’ for Long Covid, saying “oh you look better today”. However, as P1 explains, for healthcare professionals who listened, validated, and gave non-judgmental space to share experiences, the impact was profound:

One thing that stood out is the difference in health professionals’ attitudes. My GP, I think of as an exemplar. He has been caring and patient but also incredibly honest. He would say, ‘I haven't got the faintest idea what's happening to you or how to help you’, certainly in the early days, ‘but I'm going to keep monitoring you so that we can pick up any unusual things’.

This theme shows the near impossibility of navigating interactions with ‘others’ successfully unless there is belief that a person’s Long Covid is real. Negative interactions influenced psychological and social wellbeing through increased helplessness, isolation, and reducing hope for recovery.

#### A rollercoaster of psychological ambiguity

Physical symptom fluctuations and the dis/satisfaction from social encounters attributed to the fluctuating mood and wellbeing experienced by participants. Feelings of control and loss of identity were key contributors to their psychological rollercoaster. Compounded by an inability to ‘will away’ symptoms, participants described a strangeness and disconnection with their bodies, not quite knowing what the next day would bring. These sensations, and reduced participation in activities which made them feel like themselves (e.g., social activities or work), only exacerbated their loss of identity. Physical symptoms meant sacrificing experiences which previously would have given pleasure (e.g., rejecting social invitations). Sacrifices were spoken of with guilt, grief, and loss, as participants were unable to fulfil their previous, family, community, and societal roles:

One year later, that’s when I felt a great sense of loss. It hit me - “you’re not going to solve this” and “you can’t bargain to be better in a year” and so it’s a sense of loss and helplessness. My loss was going back to work. I couldn’t tell people a time I could come back, so I had to leave. There was a loss of anything I might say about myself to someone - “I’m someone who likes walking, who likes music” - all those things I said when I described myself. A complete loss of everything that made me who I am. I was still a daughter, and a mother, but I couldn’t do them in the way that I judged myself. (P7)

Without knowledge to underpin recovery expectations, some participants appeared to engage in a process of personal ‘bargaining’. For instance, they could be unwell for the rest of the year, but after that, they would be recovered. In the short term, this helped participants’ mood. Yet, long term, when expectations were not met, fear and grief returned as they remained unable to do purposeful activities. Grieving their normality, and when symptoms inhibited all meaningful participation, participants described how they felt left with a diminished life:

It makes life very small and shrunken. Physically and mentally, I feel diminished and reduced, but your whole life is like that. In between the anxiety and the stress and the worrying about the future, you end up living this very boring and dull life because you haven't got the energy to do anything. Sometimes I just sit and stare at the wall because I haven't got the effort to concentrate on something. It's like living in sludge or quicksand, an incredibly depressing way to live (P1).

Attempts to manage symptoms also contributed to their psychological rollercoaster, able to invoke both fear (e.g., if unsuccessful) and increase confidence (e.g., if successful). If strategies worked, participants described a feeling of control and openness to try further ideas. However, as P12 describes, trying new strategies could provoke dilemma and worry, given the desperate desire not to feel worse:

There is always that danger you can make yourself worse. Your decisions and activity can strain your body so there is that always at the back of your head. It’s that ‘I want to exercise, and my muscles will feel stronger, but does that put me more at risk of getting rougher?’. How do you judge that? You really want to have a medical professional holding your hand with it, because it feels like you’re taking a risk when you decide to do something.

Overall, participants’ emotional wellbeing was influenced by their perceived control of physical symptoms, and the success (or not) of interactions and personal strategies. With day-to-day encounters able to trigger feelings of deterioration (e.g., disbelief from others), and/or feelings of satisfaction (e.g., re-building identity), maintaining wellbeing was delicate. By using creative and personal strategies, some described ‘living better’ with Long Covid.

#### Sensitising mechanism: the hope-acceptance relationship

Prevalent in participants’ stories were the concepts of hope and acceptance, depicted as hope for a ‘cure’ whilst ‘not giving up’. For many, such as P5, possessing hope for finding successful strategies and full recovery, were integral to managing:

The main thing is believing that you can get better. I think if you’re sceptical and super-worried because there’s no evidence of people getting better, that just creates extra symptoms. That’s immediately going to like trigger like a negative response in your brain.

Hope was also met with apprehension. Individuals who had lived with the condition for several years, suggested hope, in the form of willing symptoms away (and pushing through), had been unhelpful. They revealed how, over time, an acceptance of their condition displaced hope. For some, this was a slow realisation that there “was no wand to be waved, or no one pill” (P6). The point of acceptance could mark a helpful ‘turning point’ for those that experienced it:

The turning point for me was acceptance and that came at around month seven. I just thought, ‘okay I am where I am, I've got to stop pushing. I've got to stop trying to find answers’ and that doesn't mean you ignore what's out there, but I stopped searching for answers that weren't there right now. I thought, ‘I've got to learn to live and accept me for how I am now. It won't be forever, but I've just got to ride it’. (P4)

Acceptance in this context amounted to increasing recognition that challenging days could happen, and there may not be a cure in the immediate future. Although a difficult shift in thinking, removing mental energy invested in active, and short-term hope created more time and space for participants to focus on strategies to live better with symptoms. An acceptance of the present did not remove hope for the long-term future, but appeared to enable participants to focus on learning and managing day-to-day symptoms, whilst waiting for medical science to find an answer. P7 shared her advice about accepting the uncertainty:

Just write ‘I am’ and leave a gap, and just let that gap be. Let that uncertainty be. I don’t know who I am exactly now, but ‘I am’ is enough. I have that written on my fridge. The end of that sentence is an adventure and doesn’t have to be what it was before. It’s probably not going to be everything it was before because I will make some changes even if I get 100%. After that, I feel more acceptance that life has changed.

For people at other stages in the ‘journey’ (e.g., a few months in), the need to actively try to recover, and accept they were doing all they could, even if unintentionally exacerbating symptoms, was integral to maintaining hope:

I’m struggling with not being a master of my own destiny. . . I wanted a plan of ‘this is what we’re going to do this week, and the improvement we’re expecting”, as pathetic as it sounded, and I can never get that. I said to [the physio], “we’re going to put together a programme. You’re going to have me back to work in three months”, and she said, “if I do that, I’ll get the Nobel Prize” [laughs]. Even though I know she can’t, that’s still the only thing that gives me hope. (P15)

This mechanism illustrated how hope and acceptance played roles in participants’ day-to-day psychological wellbeing and experiences, and acted as a lens through which their strategies were viewed.

#### Sensitising mechanism: fragile progress and being ‘better’

Perceptions of ‘progress’ and ‘better’ played a role in participants’ psychological wellbeing, their use of personal symptom-management strategies and perceptions of living ‘better’. Progress was often implicitly assumed to be a reduction in symptom prevalence and severity, feeling closer to ‘normal’. With small markers of progress came greater hope, but whilst providing some forward momentum, progress was fragile. With progress often non-linear for participants, improvements could easily be overshadowed by further change or deterioration. Progress was regarded as too small to notice day-to-day, or week-by-week, making it challenging to experience feelings of success on a day-to-day basis, and conceptualising over longer periods of time could feel more satisfying:

I’ve had this a b****y long time. Most people say, “it’s been a month, two months and I’m still no better”. No, you won’t be. In six months, you will, but you don’t look back six months. It’s so gradual the improvement, you don’t notice until something happens to surprise you. It’s like watching your own kids grow up, you don’t realise that your two-year-old has suddenly turned into a six-foot brute. If you asked me when I could see milestones in getting better, I couldn’t because they’re all so small (P3).

Beyond progress, stability also provided a sense of confidence to participants. Through developing their understanding of triggers and symptoms, and the trial-and-error of personal symptom-management strategies, symptom stability (e.g., a plateau) could instil feelings of control and confidence, and make them feel ‘better’. Similarly, a shift in participants perceptions of being ‘better’ appeared to influence their day-to-day mood. For those able to ‘live better’, ‘better’ was not always attributed to change or reduction in physical symptoms. It was felt to encompass fewer feelings of guilt or anxiety, or greater participation in meaningful activity. P6 described how the value of being ‘better’ for moments, and recognising and maximising those pockets of ‘better’, could produce a cumulative effect:

People would say “are you better?”. At first, it used to anger me because I thought “no, I’m not better, I’m not running, I’m not doing this”, but then I thought… It’s, my understanding of what is meant by better, and actually, some days were better than others. I didn’t know why, but throughout the day, certain hours could feel a bit better. Other times admittedly, I could feel worse, but then it was around thinking about things that could increase more of those better moments.

Overall 'progress' and 'being better' were not merely considered to be long term symptom improvements. Instead, those who could feel ‘better’, considered symptom control and stability as progress, and could begin to recognise moments of pleasure even if very brief.

### Personal strategies to manage everyday life

Participants narratives illustrated an inability to do nothing which fuelled a proactive desire to search for ideas and curate strategies according to their own unique symptoms and contexts. The theme is constructed of five sub-themes which illustrates the development and value of personal strategies. These are depicted in a temporal flow, whereby participants tended to speak of sub-themes (e.g., *seeking re-assurance and knowledge*) earlier on in their experience, and sub-themes (e.g., *prioritising ‘me’*) as knowledge, acceptance and understanding of their condition grew.

#### Seeking re-assurance and knowledge

Initially, participants spoke of engaging in strategies to make sense of their experiences. For instance, during the diagnosis or realisation of Long Covid, medical answers (e.g., from X rays, MRI scans and blood tests) were sought. Medical advice could both increase (e.g., if nothing found) or decrease (e.g., if issue identified) fear and uncertainty, while care and helpful information could instil tentative hope for recovery.

When medical services were not available, participants sought advice from alternative sources. With the emergence, and recognition of Long Covid, came websites and social media pages for the community (e.g., Long Covid Support). Participants shared how online information provided reassurance and validation, and was helpful for learning about the condition, and explaining symptoms to others. However, navigating and knowing what sources to trust was difficult and time consuming. For some, social media was considered distressing with some stories perpetuating feelings of helplessness and fear. However, for others, such as P13, carefully navigating social media also had perks:

I would say, do read stuff, do look at social media, do look at the medical journals. You can feel a bit of control of what’s going on, but you have to take that with a pinch of salt. It’s kind of like a fine balance.

Here, knowledge of Long Covid could be powerful and comforting, but energy intensive to source, and challenging to sustain. However, the process of searching could provide feelings of purpose, and in the absence of medical services, gave participants an active role in their own recovery.

#### Developing self-awareness of symptoms through monitoring

Participants considered time spent reflecting on personal symptom prevalence, triggers, patterns, and their impact, a valuable investment of energy. To enhance self-awareness of symptoms, various monitoring strategies and equipment were used (e.g., mind-maps, wall charts, diaries, smart watches). Participants described how monitoring symptoms could facilitate a deeper understanding of their personal condition and aid them in selecting and adapting existing strategies. With time, they described recognising symptom triggers, and this learning, and subsequent feelings of control, were considered progress:

What I’ve found is helping is not to get to that [crash] point. I’ve found correlations where if I do certain activities, I’m more susceptible to getting right down… For me, I call it the eye of the storm. If the fatigue is the eye, everything else comes around it. If I can minimise the fatigue, everything else is not as bad. For other people, the eye might not be fatigue, it might be a different symptom, but that’s where I think a log comes in. It’s being able to track and think, ‘okay, well it looks like everything gets worse when that symptom is at its worst so is there anything I can do to minimise that initial symptom?’. (P2)

Patterns between symptoms differed from person to person. P10 noted how his Long Covid was “entirely linear”; he could predict his fatigue from one day to the next based upon his activities. For others, patterns could remain fuzzy and hard to predict. Regardless, participants’ confidence appeared to grow with greater knowledge of their personal symptoms, triggers, and connections. Personal knowledge further facilitated communication of their illness to others.

#### Trial-and-error of ‘safe’ ideas

Participants explained developing and implementing different strategies to manage symptoms. Across the banks of strategies described, a commonality was the ‘safe’ nature of ideas. For instance, ideas which were not considered harmful (e.g., medication), or be likely to deteriorate symptoms. Implementation resembled trial-and-error, by trying, observing any effect, and then either trying things differently, or instigating the next idea.

Multiple strategies were experimented with by all participants. For fatigue, these included pacing, prioritising, preventing heart rate increases and rest (including micro-rests). Television and reading could be too cognitively stimulating. Instead, yoga, lying down in silence and meditation were considered more restful activities. For cognitive challenges, ideas included avoiding screens, being in dark spaces, and using smart devices for reminders. Dietary changes (e.g., vitamins, supplements, low-histamine and anti-inflammatory foods, caffeine intake), inhalers and breathing exercises were also amongst the countless ideas tried. Strategies, such as pacing, were approached uniquely by participants. P2 described pacing using “a jar of sugar cubes” to visually display her energy and physically take cubes out following activity, whereas P4 explained a slow-motion technique where activities were completed but much slower. These differences highlighted the importance of personal preference and the value of finding what worked for them.

Throughout the trial-and-error process, participants expressed the need to be open to new ideas and adaptations of past ideas. It was expressed that such openness could enhance the likeliness of finding successful solutions and appeared to foster greater feelings of control and less of helplessness. With ‘normal’ now unknown, adaptations to existing taken-for-granted habits were also suggested as P12 described:

I would always say ‘I can’t sleep in the day’ and that’s still true, but some people may find that something isn’t true anymore. I always had a certain pillow, and now that pillow doesn’t work anymore, so your sleep is affected. All that time, you think, ‘I know what works for me’ but maybe it doesn’t. You need to experiment, even if you’re sure that pillow or tog of duvet is right for you. I never used to sleep with the window open and now I do. Things that I thought were sorted have been completely turned on their head, so you need to be open to your body just wanting different things to before.

Overall, the trial-and-error process impacted participants’ emotional wellbeing and physical symptoms. Success of personal strategies appeared to enhance confidence and mood, while unsuccessful ideas could prompt distress, grief, a loss of control and diminished hope. With evolving symptoms, some strategies were maintained while others replaced. Such instability meant feelings of symptom control were flimsy, as strategies could stop working at any time. However, trial-and-error did provide opportunities for enhancing symptom knowledge, conceptualised by some as progress.

#### Building in pleasure and comfort

Given the difficulty in controlling symptoms, some participants prioritised strategies that enabled participation in meaningful and joyful activities. P7 described how these pleasurable moments were crucial amid uncertainty: “time enjoyed is never time wasted and joyful moments are essential parts of the day”. With hope of a return to ‘normality’, participants tried to re-engage and reconnect with themselves. Some had to adapt activities, or do them differently, to make them achievable. For instance, sitting instead of standing, using a wheelchair, implementing rest breaks, and doing activities at home, instead of venturing out. Joy was not considered something that needed to be ‘perfect’, but something to be squeezed from any possible moment to try and feel better:

I try to extract as much joy as possible out of the small things that you take for granted. The Ashes has just started, I get a lot of insomnia, I'm awake in the night and at least I can watch the cricket. Or when they started playing football again, just having a football match to look forward to or a TV show that's come back… that kept me going, trying to just appreciate small victories. I still cook and I still have my wife, and I put a bit of effort into that, and it makes her happy and makes me a bit happy. Life gets smaller but try to enjoy the little wins as much as possible. (P1)

Similarly, there was an openness to new pleasurable activities that didn’t trigger symptoms, including crafting, podcasts, and yoga. Some participants expressed surprise and even gratitude for these new experiences. With time to stop and reflect, opportunities arose for some that might not have emerged. These included career changes and new social opportunities (e.g., forging new friendships through Long Covid communities on social media). When planning joyful activities, participants considered flexibility vital (e.g., creating Plan B and C). This was to ensure participation could be undertaken in some capacity, not to disappoint themselves or others.

Connections with friends, family, and colleagues who would listen provided comfort and support; the caveat being that these individuals had to believe the condition was real and demonstrated that through action. Participants sought support from these individuals where they could best find it (e.g., not necessarily previously close social ties), and unsupportive relationships were fizzled out. Described as ‘phasing out drains’, this was a strategy of self-protection to maintain emotional wellbeing and save energy:

For one person to knock you down is not fair, so you have to protect yourself. I developed a barrier between myself and everyone else, and how much I talk about it…You need to have a safe space. My mum, dad, and best friend, they’re the only people I go to because I know they’ll support and believe me. (P2)

Although communication could be energy-consuming, strategies such as WhatsApp, and emojis allowed interaction at times that suited participants, sometimes without needing words. Engaging with trusted social ties, and meaningful activity provided social and emotional fulfilment and helped participants feel more like themselves again.

#### Prioritising ‘me’

Participants’ unique presentations of Long Covid meant a need to prioritise themselves before anything else. Trying to push through and feeling pressured to do so (e.g., by employers/family), was not helpful. Yet, prioritising, trying new strategies, adapting old habits and ‘being kind to yourself’ could also attract feelings of guilt. For some participants, prioritisation had to be in-keeping with their social roles. For instance, as P8 expressed, first-aid kit symptom-management strategies were used to balance responsibility and personal prioritisation:

As a busy working mum with young children, you put yourself last all the time. I realised that was the big shift I needed to try to get better. On the outside, put everyone first, but have moments where I check my smart watch or have that moment of mindfulness, and check in with myself. That might be enough to drop my heart rate, help start the recovery and stop the cycle. It’s like putting a plaster on the wound really, but it would just be enough to keep going for a bit longer.

Prioritisation could include employment changes (e.g., extending leave, permanently leaving). Although this removed a part of their personal identity, engaging in a cycle of working, crashing, feeling better, working, and crashing again only enhanced the emotional rollercoaster experienced. Listening to their bodies, trusting personal instincts, learning symptom triggers, and learning symptom-management strategies comprised the main strategies for prioritisation. As P4 mentioned “you have to accept what your body is telling you”. The importance of personal prioritisation for living with Long Covid was perceived as a clear and active step towards recovery, but this looked different for everyone. Overall, the personal self-generated strategies to live better tended to instil feelings of confidence, sensations of progress and help regain some control over their diminished lives.

## Discussion

### Summary of findings

This paper illustrates the complex day-to-day experiences and the number of rich and creative self-generated strategies used by people living with Long Covid. Participants all described how their lives with Covid-19 and subsequent Long Covid had been shaped by the wider social context. Experiences were characterised by fluctuating physical symptoms which inhibited participation in everyday activities. This was depicted as an interconnected web of social, physical, and emotional challenges. This study adds further clarity into creative, novel but above all personal strategies used to navigate the condition and live day-to-day.

### Comparison with previous literature

Showing similarity with other studies, social and emotional challenges were attributed to physical symptoms [10, 11]. Here, physical symptoms were described using metaphorical language to emphasise severity [33]. Termed an episodic disability [10], Long Covid physical symptoms were unpredictable, and feelings of hope and progress were fragile. Conceptualised as ‘disrupted chronology’ [34] or ‘narrative wreckage’ [35], people’s future trajectories were suddenly interrupted, and such disruption, alongside constant physical challenges, induced strong emotional responses such as grief, guilt, fear, and frustration [10, 12, 13, 15]. As O’Brien and colleagues outlined, fear was not only episodical (e.g., fear of symptom severity the following day), but also longitudinal (e.g., fear of the ambiguity surrounding full recovery) [10], contributing to a continuous, perplexing impact on their wellbeing. Aligned with other studies [12, 14, 15, 17, 21], diminished capabilities left people feeling unlike themselves, and questioning their identity and purpose in life.

As in other qualitative studies, the invisibility of Long Covid created challenges during social interaction [10, 17]. For instance, Callan and colleagues also found that people with ‘brain fog’ symptoms were not taken seriously by healthcare professionals due to an inability to verbally convey symptoms (e.g., lack of preparedness) [11]. However, the present findings extend knowledge by indicating that being too prepared, and appearing ‘too well’, can also lead to disbelief, gaslighting and a lack of validation. Although Ladds and colleagues found that creative methods were required to access healthcare services, such as ‘playing the game’ (e.g., only sharing specific information), and calling on friends and family for ‘back door’ appointments [9], the present study, like past studies [9, 36], suggests that the main barrier to healthcare provision, may be healthcare professionals’ attitudes to Long Covid. With individuals now risking economic burden by accessing private healthcare provision [13], our findings suggest such consultations will not provide any greater support unless those healthcare practitioners believe and validate their Long Covid experiences.

As suggested by Pearson et al., social contexts, such as public perception, also influenced Long Covid experiences [17]. Political in/activity associated with the management of Covid-19 led people to feel stigmatised for having Covid-19 and Long Covid, and this feeling was lasting. Although Long Covid had been officially recognised at the time of interviews [1], instead of situating blame with friends and families lack of understanding, fault was directed towards ‘higher powers’, including government and national media for the limited exposure and denial of the condition’s substantive nature. Although the political and health climate has evolved since the undertaking of these interviews, people with Long Covid continue to feel stigmatised, transitioning from ‘poor, unlucky victims’, to now ‘weak and anxious malingerers’.

Key constructs that featured throughout day-to-day Long Covid experiences were hope, acceptance and progress. Hope for immediate improvement and recovery appeared to feature in the earlier stages of Long Covid, but with time, and in those who considered themselves to be living better with their Long Covid, a form of acceptance replaced this. Drawing from the work of Ratcliffe, it may be that to live better with the condition, a person must retain a different kind of hope [37]. For instance, people with Long Covid may lose hope of immediate improvement but retain hope of a future full recovery. As Ratcliffe explains, pre-intentional hope (e.g., attitude towards full recovery), may survive the loss of any intentional hopes (e.g., immediate improvement) that are being actively sought out [37]. For people with Long Covid, a combination of present acceptance but hope for the future may represent their personal strategy to navigate day-to-day activities.

Findings further illustrate novel perspectives of ‘progress’ and being ‘better’ when living with Long Covid. Noted in previous research, ‘better’ is often conflated with recovery or resolution [10]. Instead of conceptualising ‘better’ as symptom reduction and as a more static entity, some participants in this study recognised ‘better’ as a facet that could be ambivalent to symptoms, and fluid, whereby they could feel better for small moments throughout a day. Similar assumptions can logically be made about the term ‘progress’, which is considered any action towards an end outcome. Drawing from illness narratives of restitution, quest, and chaos [35], these trajectories present outcomes of recovery or cure, new transformation following illness, or ongoing incoherence and despair, respectively. However, unlike these narratives, some people with Long Covid described wanting a path of stability, gaining some control over symptoms, and subsequently more able to navigate their everyday activities. Previously, O’Brien and colleagues described “an absence of a progressive path from illness to wellness” (p.6) for people with Long Covid [10]. This is problematic given the only trajectory remaining is unknown and chaotic. Therefore, a trajectory of stability may provide an alternative, viable option for people with Long Covid, and an alternative path for people to make sense of their experiences, gain feelings of inclusivity and prevent isolation and silence [38].

Consistent with previous literature, multiple strategies were tried to self-manage symptoms and live better with Long Covid. These included, but were not limited to, dietary changes [11, 21, 39], fatigue management strategies [10–12, 17], peer support [13], and symptom distraction [21]. The need to build joy into day-to-day life was also key to living better with Long Covid. As reported elsewhere, small pockets of pleasure provided relief and distraction from debilitating symptoms [13, 21], and could help establish a sense of identity and renewed purpose. While previous research provides a plethora of possible strategies, the present paper extends knowledge to illustrate the self-generated, self-management strategies in greater depth. Specifically, the findings highlight the temporal nature of self-management and the importance of the time and energy investment to reflect upon and understand each person’s experiences in depth (e.g., considering all symptoms and triggers together). By looking at symptoms together as a whole, rather than individually, participants in the study described a greater sense of control and awareness. O’Brien et al. describe this process as a form of uncertainty management [10]. Here, enhancing a person’s self-awareness of their condition could evoke greater feelings of predictability and confidence. Although adjusting to the uncertainty of Long Covid can take time [17], strategies to enhance self-awareness, and actively learn about symptoms enabled feelings of control and greater day-to-day purpose. This holistic self-directed learning helped to develop and implement strategies based upon each participant and their unique experiences, and highlighted the importance of finding what works for the person. These findings support the need for continued attention to person-centred care by healthcare professionals working in Long Covid, and the inevitable shortcomings of generic, prescriptive advice.

Ultimately, our paper supports the recommendation that self-management support should be tailored to individuals’ abilities, interests, and needs, whilst recognising the complex interplay of Long Covid symptoms [10, 21, 40]. Only by giving a person time and space to share their complexities and challenges, can the multiple trajectories beyond recovery and chaos, be fully understood. However, to provide reassurance and to facilitate symptom sense-making, rehabilitation and self-management support should be available alongside timely medical investigations to fully understand each person’s underlying Long Covid pathophysiology. In combination with ideas and priorities generated from the wider co-design process, these findings have informed the development of the LISTEN intervention which is being evaluated as part of a clinical trial and compared to usual NHS Long Covid services [41]. The stories of participants comprise a narrative-based resource, and findings have been incorporated into a training package for nurses and allied healthcare professionals delivering the intervention. The findings of the LISTEN trial will be published upon completion.

### Strengths and limitations of the study

A significant strength of this study was the inclusion and contribution of people with living Long Covid in the data analysis and write up. Enhancing the transparency of the research process, and trustworthiness in data interpretation, authors living with Long Covid supported authors without lived experience to portray findings as authentically as possible. It is hoped that using a visual illustration to display the interrelation between themes, and using everyday terminology where possible, will support the Long Covid community to access and connect with the findings of our research.

A limitation of this research was the sample, which may not fully reflect the diversity the Long Covid community, despite multiple strategies for recruitment [41]. Participants were those able to access and participate in co-design activities held during traditional working hours. Consequently, we may not have captured the views of the people with Long Covid who were unable to share their valuable energy and time with us. This may influence the transferability of findings such as the theme *prioritising ‘me’*. Additionally, participants had already participated in several LISTEN co-design activities, which might have shaped ‘how to talk about Long Covid’ by the time they reached the interview stage. Stories were therefore told with relative fluidity which may not be reflected in the wider community. Furthermore, participants were only recruited from England and Wales, and as such the international transferability of our findings are unknown. Across the globe, Long Covid has attracted differing media attention and government policies, but the experiences shared within this manuscript focus on a specific population at a specific time. Alluded to throughout the paper, the context around Long Covid is constantly evolving, and attention needs to be given to the context and timing of the condition, as well as each individual’s personal story.

## Conclusion

In summary, strategies used to manage everyday life with Long Covid comprised personal solutions, unique to individuals’ symptoms, surroundings, and experiences. Physical symptoms impacted psychological wellbeing, and therefore managing everyday life was not solely focused on symptom reduction, but also finding enjoyment and a sense of identity. These results add to the understanding of the lived experiences of adults living with Long Covid in England and Wales, illustrate the impact of the wider Covid context on individuals’ experiences and provide an overview of self-generated strategies that could be used by people living with Long Covid and healthcare professionals who support them.

### Supplementary Information

**Supplementary Material 1.**

## Data Availability

The dataset generated and analysed during the current study are not publicly available due to lack of participant consent for this, but an anonymised dataset may be available from the corresponding author on reasonable request.

## Footnotes

1 Some participants felt the term ‘journey’ did not adequately represent their experience. Instead, they felt like they had neither made progress backwards or forwards, and thus described their experience as stuck, and static, rather than a journey which implies movement.

## References

[1] World Health Organisation (WHO). A clinical case definition of post COVID-19 condition by a Delphi consensus. 2021. https://apps.who.int/iris/bitstream/handle/10665/345824/WHO-2019-nCoV-Post-COVID-19-condition-Clinical-case-definition-2021.1-eng.pdf. Accessed 28 Jul 2022.

[2] National Institute for Health and Care Research (NIHR). Living with Covid19 – Second review. 2021. 10.3310/themedreview_45225. Accessed 28 Jul 2022.

[3] Callard F, Perego E (2021). How and why patients made Long Covid. Soc Sci Med.

[4] Office for National Statistics (ONS). Prevalence of ongoing symptoms following coronavirus (COVID-19) infection in the UK. 2023. https://www.ons.gov.uk/peoplepopulationandcommunity/healthandsocialcare/conditionsanddiseases/bulletins/prevalenceofongoingsymptomsfollowingcoronaviruscovid19infectionintheuk/30march2023. Accessed 4 May 2023.

[5] Davis HE, Assaf GS, McCorkell L, Wei H, Low RJ, Re’em Y, et al. Characterizing long COVID in an international cohort: 7 months of symptoms and their impact. EClinicalMedicine. 2021;38:101019. 10.1016/j.eclinm.2021.101019 PMC8280690 34308300

[6] British Medical Association (BMA). Addressing the health challenge of long COVID. 2022. https://www.bma.org.uk/media/6056/addressing-the-health-challenge-of-long-covid-final.pdf. Accessed 4 May 2023.

[7] Office for National Statistics. Prevalence of ongoing symptoms following coronavirus (COVID-19) infection in the UK. 2022. https://www.ons.gov.uk/peoplepopulationandcommunity/healthandsocialcare/conditionsanddiseases/bulletins/prevalenceofongoingsymptomsfollowingcoronaviruscovid19infectionintheuk/7july2022. Accessed 4 May 2023.

[8] Aiyegbusi OL, Hughes SE, Turner G, Grace R, Cruz S, McMullan C (2021). Symptoms, complications and management of long COVID: a review. J R Soc Med.

[9] Ladds E, Rushforth A, Wieringa S, Taylor S, Rayner C, Husain L, Greenhalgh T (2020). Persistent symptoms after Covid-19: qualitative study of 114 “long Covid” patients and draft quality principles for services. BMC Health Ser Res.

[10] O’Brien KK, Brown DA, Mcduff K, St Clair-Sullivan N, Solomon P, Chan C (2023). Conceptualising the episodic nature of disability among adults living with Long COVID: a qualitative study. BMJ Glob Health.

[11] Callan C, Ladds E, Husain L, Pattinson K, Greenhalgh T (2022). ‘I can’t cope with multiple inputs’: a qualitative study of the lived experience of ‘brain fog’ after COVID-19. BMJ Open.

[12] Macpherson K, Cooper K, Harbour J, Mahal D, Miller C, Nairn M (2022). Experiences of living with long COVID and of accessing healthcare services: a qualitative systematic review. BMJ Open.

[13] Burton A, Aughterson H, Fancourt D, Philip KEJ (2022). Factors shaping the mental health and well-being of people experiencing persistent COVID-19 symptoms or ‘long COVID’: qualitative study. BJ Psych Open.

[14] Wurz A, Culos-Reed SN, Franklin K, DeMars J, Wrightson JG, Twomey R (2022). I feel like my body is broken: exploring the experiences of people living with long COVID. Qual of Life Res.

[15] Ireson J, Taylor A, Richardson E, Greenfield B, Jones G (2022). Exploring invisibility and epistemic injustice in Long Covid—A citizen science qualitative analysis of patient stories from an online Covid community. Health Exp.

[16] Reuschke D, Houston D (2022). The impact of Long COVID on the UK workforce. Applied Economic Letters.

[17] Pearson M, Allsopp G, Bartel H, Singh P, Crawford P (2022). Creative Long Covid: A qualitative exploration of the experience of Long Covid through the medium of creative narratives. Health Exp.

[18] National Health Service (NHS) England. National commissioning guidance for post COVID services. 2022. https://www.england.nhs.uk/publication/national-commissioning-guidance-for-post-covid-services/. Accessed 4 May 2023.

[19] Living with Ltd. Living with Covid Recovery. 2023. https://livingwith.health/covid-recovery/. Accessed 28 Nov 2023.

[20] National Health Service (NHS) England. Your COVID Recovery. 2022. https://www.yourcovidrecovery.nhs.uk/. Accessed 28 Nov 2023.

[21] Loft MI, Foged EM, Koreska M (2022). An Unexpected Journey: The Lived Experiences of Patients with Long-Term Cognitive Sequelae After Recovering from COVID-19. Qual Health Research.

[22] Wright H, Turner A, Ennis S, Percy C, Loftus G, Clyne W, Matouskova G, Martin F (2022). Digital Peer-Supported Self-Management Intervention Codesigned by People with Long COVID: Mixed Methods Proof-of-Concept Study. JMIR Form Res.

[23] National Institute for Health and Care Research (NIHR). £19.6 million awarded to new research studies to help diagnose and treat long COVID. 2021. https://www.nihr.ac.uk/news/196-million-awarded-to-new-research-studies-to-help-diagnose-and-treat-long-covid/28205. Accessed 4 May 2023.

[24] Locock L, Robert G, Boaz A, Vougioukalou S, Shuldham C, Fielden J (2014). Testing accelerated experience-based co-design: a qualitative study of using a national archive of patient experience narrative interviews to promote rapid patient-centred service improvement. Health Ser Deli Res.

[25] Robert G, Cornwell J, Locock L, Purushotham A, Sturmey G, Gager M (2015). Patients and staff as codesigners of healthcare services. BMJ.

[26] Heaton-Shrestha C, Torrens-Burton A, Leggat F, Islam I, Busse M, Jones F (2022). Co-designing personalised self-management support for people living with long Covid: the LISTEN protocol. PLoS ONE.

[27] Braun V, Clarke V (2019). Reflecting on reflexive thematic analysis. Qual Res Sport Exercise.

[28] Braun V, Clarke V (2006). Using thematic analysis in psychology. Qual Res Psych.

[29] Braun V, Clarke V (2021). Thematic analysis: a practical guide.

[30] Smith B (2018). Generalizability in qualitative research: misunderstandings, opportunities and recommendations for the sport and exercise sciences. Qual Res Sport Exercise.

[31] Richardson EV, Motl RW (2021). “Kicking and Screaming” or “Gracefully Conceding”: Creative Nonfiction Stories of Aging With Multiple Sclerosis. Qual Health Res.

[32] Smith B, McGannon KR (2018). Developing rigor in qualitative research: problems and opportunities within sport and exercise psychology. Int Rev Sport Exercise Psych.

[33] Chasco EE, Dukes K, Jones D, Comellas AP, Hoffman RM, Garg A (2022). Brain Fog and Fatigue following COVID-19 Infection: An Exploratory Study of Patient Experiences of Long COVID. Int Jour Env Res Pub Health.

[34] Rushforth A, Ladds E, Wieringa S, Taylor S, Husain L, Greenhalgh T (2021). Long Covid – The illness narratives. Soc Sci Med.

[35] Frank AW (2013). The Wounded Storyteller.

[36] Ladds E, Rushforth A, Wieringa S, Taylor S, Rayner C, Husain L, Greenhalgh T (2021). Developing services for long COVID: lessons from a study of wounded healers. Clin Med (London).

[37] Ratcliffe M (2013). What is it to lose hope?. Phenom Cog Sci.

[38] Richardson EV, Motl RW (2020). Promoting Inclusion in a Fitness Centre through Non-Impaired Staff: Creating a Multi-Narrative Environment. Qual Res Sport Exercise Health.

[39] Kingstone T, Taylor AK, O’Donnell CA, Atherton H, Blane DN, Chew-Graham CA (2020). Finding the “right” GP: a qualitative study of the experiences of people with long-COVID. BJGP Open.

[40] Bulley C, Tyagi V, Curnow E, Nicol K, Salisbury L, Stuart K, et al. Support after COVID-19 study: a mixed-methods cross-sectional study to develop recommendations for practice. BMJ Open. 2022;12:e056568. 10.1136/bmjopen-2021-056568 PMC9437739 36038169

[41] Potter C, Leggat F, Lowe R, Pallmann P, Riaz M, Barlow C (2023). Effectiveness and cost-effectiveness of a personalised self-management intervention for living with long COVID: protocol for the LISTEN randomised controlled trial. Trials.

