# Supplementary material for: An exploration of the experiences and self-generated strategies used when navigating everyday life with Long Covid
Source: BMC Public Health. 2024 Mar 13;24:789. doi: 10.1186/s12889-024-18267-6 (PMC10938753; doi:10.1186/s12889-024-18267-6)
Supplement: Supplementary file 1 — Supplementary Material 1. [file 12889_2024_18267_MOESM1_ESM.pdf]

Kingston University London
St George's University of London

GREATER +TOGETHER
Kingston and St George's

Faculty of Health, Social Care and Education
Kingston Hill Campus, Kingston Hill
Kingston upon Thames, KT2 7LB

Telephone: (0)20 8417 5000

www.healthcare.ac.uk

People living with or recovered from Long COVID Filmed Interviews – interview guide

Long COVID Personalised Self-managemenT support- co-design and EvaluatioN (LISTEN)

[Researcher: thank interviewee for their time; remind interviewee that they can take a break or pause the interview if they feel they need to; ask if they have any questions before you start the interview and clarify as far as is possible]

1. Please can you tell me a little bit about yourself to help me get to know you.
2. Can you tell me a bit about when you caught Covid-19? What was that like?
3. Can you explain how you realised you had Long Covid?
4. Can you say a little about the challenges you experienced? Can you give some examples?
5. Can you tell us about some successes you feel you have had, living and working with Long Covid? Can you describe these?
6. What have you found helpful to navigate life with Long Covid?
7. What would you like to say to others living with Long Covid?
